# Subfertile patients underestimate their risk factors of reprotoxic exposure

**DOI:** 10.1186/s12610-022-00161-z

**Published:** 2022-07-05

**Authors:** Nadia Nouiakh, Claire Sunyach, Sarah-Lyne Jos, Irène Sari-Minodier, Catherine Metzler-Guillemain, Blandine Courbiere, Florence Bretelle, Jeanne Perrin

**Affiliations:** 1 Centre Clinico-Biologique d’AMP-CECOS, AP-HM La Conception University Hospital, 147 bd Baille, 13005 Marseille, France; 2 Plateforme CREER, AP-HM La Conception University Hospital, 147 bd Baille, 13005 Marseille, France; 3 Service de Médecine et Santé au Travail, AP-HM La Timone University Hospital, 145 rue St Pierre, 13005 Marseille, France; 4 Aix Marseille Univ, Avignon Université, CNRS, IRD, IMBE, 27 bd J Moulin, 13385 Marseille, France; 5 Aix Marseille Univ, Inserm, MMG, U1251, Marseille Medical Genetics, 27 bd J Moulin, 13385 Marseille, France; 6 Aix Marseille Univ, IRD, AP-HM,MEPHI, IHU Méditerranée Infection, Marseille, France

**Keywords:** Environmental exposure, Couple infertility, Male fertility, Occupational exposure, Assisted reproductive technique, Man’s health, Expositions environnementales, Couple infertile, Expositions professionnelles, Assistance médicale à la procreation, Santé masculine

## Abstract

**Background:**

Exposure of men and women to environmental reprotoxic agents is associated with impaired fertility and pregnancy rates after assisted reproductive treatment (ART). Nevertheless, such exposures are generally not systematically assessed in current practice before ART and subfertile men are generally less explored than women. Our objective was to study subfertile men and women’s level of knowledge about reprotoxic agents, their perception of their own risk factors and the correlation between perceived and identified circumstances of exposure.

**Results:**

In our public university hospital, 390 subfertile patients (185 men and 185 women) requiring assisted reproduction technique (ART) treatment, completed a self-report questionnaire before consultation, in order to assess patients’ knowledge of reprotoxic exposures, sources of information about them and perception of their own circumstances of exposure. Then a standardized questionnaire was used by the physician during the consultation to estimate domestic, environmental and occupational risk factors of reprotoxic exposures (RFRE). We compared the patients’ perception of exposure with the estimated RFRE.

The reprotoxic agents knowledge score of patients was 61%. Their main sources of information were the media (40%), the internet (22%) and gynecologists (15%). The standardized questionnaire identified RFRE in 265/390 patients (68%); risk factor was statistically more frequent in men (77%) than in women (59%) (*p* < 0.05). In total, 141 of the 265 patients with identified RFRE (53%) were aware of their risk factor of reprotoxic exposure.

**Conclusion:**

We identified risk factors of reprotoxic exposures in the majority of subfertile patients, more frequently in men than in women, and half of patients were not aware of their exposures. Patients’ main sources of information were extra medical. Efforts should be made to inform patients, especially men, about potential reprotoxic exposure and to enhance medical training about reprotoxic agents, as recommended by international guidelines. The detection and correction of environmental exposures in subfertile men could improve their fecundity, but also their general health, which has been shown to be poorer than health of fertile men.

**Supplementary Information:**

The online version contains supplementary material available at 10.1186/s12610-022-00161-z.

## Résumé

**Contexte:**

L’exposition des hommes et des femmes à des agents reprotoxiques environnementaux est. associée à une atération de leur fertilité et des taux de grossesse après assistance médicale à la procreation (AMP). Néanmoins, ces expositions ne sont généralement pas recherchées en pratique courante avant AMP et les hommes infertiles sont généralement moins explorés que les femmes. Notre objectif était d’analyser le niveau de connaissance des hommes et des femmes infertiles sur les expositions environnementales reprotoxiques, leur perception de leurs propres facteurs de risque et la correlation entre les expositions reprotoxiques perçues et celles identifiées.

**Resultats:**

Dans notre centre hospitalier universitaire, 390 patients infertiles (185 hommes et 185 femmes) nécessitant un traitement d’AMP ont complété un auto-questionnaire avant la consultation, afin d’évaluer leurs connaissances sur les expositions reprotoxiques, leurs sources d’information sur ce sujet, et leur perception de leurs propres expositions. Puis, lors de la consultation, le médecin utilisait un questionnaire standardisé pour estimer leurs facteurs de risque d’exposition reprotoxique (FRER) domestiques, environnementaux et professionnels. Nous avons comparé la perception par les patients de leurs propres expositions reprotoxiques avec le FRER estimé par le médecin. Le score de connaissance des agents reprotoxique des patients était de 61%. Leurs sources d’information principales étaient les medias (40%), internet (22%), et les gynécologues (15%). Le questionnaire standardisé identifiait un FRER chez 265/390 patients (68%); les FRER étaient significativement plus fréquents chez les hommes (77%) que chez le femmes (59%)(*p* < 0.05). Au total, 141 patients sur les 265 avec un FRER identifié étaient conscients de leur FRER.

**Conclusion:**

Nous avons identifié des facteurs de risque d’exposition reprotoxiques chez la majorité des patients infertiles, plus fréquemment chez les hommes que chez les femmes, et la moitié des patients n’étaient conscients de ces expositions. Les principals sources d’information des patients étaient extra-médicales. Des efforts sont nécessaires pour informer les patients, en particulier les hommes, sur les potentielles expositions reprotoxiques comme souligné par les recommandations internationales. La detection et la correction des expositions environnementales chez les hommes infertiles pourraient améliorer leur fécondité, mais aussi leur santé, qui a été démontrée comme moins bonne que celle des hommes fertiles.

## Background

For subfertile patients undergoing assisted reproductive treatment (ART), the chances of the birth of a healthy child can be affected by multiple factors, such as body mass index (BMI), nutritional habits, smoking, marijuana consumption and exposure to environmental, dietary and professional pollution agents [1–7]. Indeed, epidemiologic data accumulated in recent years have shown the deleterious effects of exposure to chemical and physical environmental and professional toxic agents on reproduction, affecting male and female fertility, time to pregnancy and pregnancy development [8–12].

Since 2013, multiple international scientific societies have taken a stand favoring the identification and reduction of exposure to environmental and professional chemicals during the preconception, conception and perinatal periods [13]. Recently, Segal and Giudice proposed practical directives for professionals to interrogate and advise their patients in order to detect and decrease their exposure to reprotoxic agents [12]. Despite these developments and the multiple calls to integrate environmental health topics into consultations [13], healthcare professionals face difficulties in current practice, mainly related to a lack of knowledge and a lack of time available during infertility consultations [14, 15]. Consequently, multiple studies have demonstrated that the medical personnel caring for patients during the perinatal and gestational periods did not adequately inform them about such risks [14–16].

Another difficulty is that monitoring exposure to reprotoxic agents is complex and expensive and therefore not adapted to daily practice: reprotoxic agents are multiple in term of chemical family, metabolites, stability, half-life. Analytical techniques are available for many chemicals including persistent and non-persistent endocrine disruptors [17], but several preanalytical factors can affect sample quality for blood and serum such as special collection (tubes) and storage conditions and processing of samples. Acceptability of the biological sample collection by both couples and the healthcare teams can also hampered such study. Nevertheless, the French ELFE cohort showed that Bisphenol A and some metabolites of phthalates, pesticides (mainly pyrethroids), dioxins, furans, polychlorobiphenyls, brominated flame retardants, perfluorinated compounds and metals (except uranium) were quantified in almost 100% of the 4145 pregnant women included [18].

Importantly, some environmental reprotoxic exposures are modifiable and can be identified. Nevertheless, the knowledge and perception of subfertile patients regarding their exposure to environmental reprotoxic agents are not very well developed, as previously shown in a study suggesting that subfertile men underestimate their exposure to reprotoxic agents [19]. Subfertile patients can act on their fertility by modifying some daily life habits, as suggested by several publications showing that numerous patients undergoing assisted reproduction were eager to change their habits and wish to be counseled to decrease their exposure to reprotoxic agents [19–21]. However, to endorse daily habit and lifestyle changes, patients must be informed and fully conscious of the reprotoxic risk factors.

In this context, our study questions were: what is the knowledge of subfertile patients about reprotoxic risk factors, and from which information source? What is the perception of subfertile patients about their own risks factors of reprotoxic exposure? Do this perception correlate with the risk factor of reprotoxic exposure estimated by a physician? By comparing “perceived risk” with “estimated risk”, we aimed to investigate whether medical intervention to estimate these risks would be useful in routine practice, or whether patients estimate them well on their own. To circumvent the difficulty of assessing reprotoxic exposures by quantitative biomarkers, we assessed risk factor of reprotoxic exposure using a standardized questionnaire in the current practice of our infertility center.

## Materials and methods

### Population

In this prospective observational study, we included all the patients who visited our fertility unit and consented to participate between March 2016 and November 2017.

The inclusion criteria were as follows: all men between the ages of 18 and 58 years and all women between the ages of 18 and 43 years, visiting the fertility unit for a consultation before starting ART. The exclusion criteria were patients who did not master French language.

### Questionnaire content

Prior to the medical consultation, the man and woman of each couple completed a questionnaire (Additional file 1) that was divided into four segments: 1) general information about each patient (sex, age, source of information about reprotoxic agents); 2) participants’ perception of their personal exposure to reprotoxic agents; 3) participants’ knowledge of reprotoxic agents they may be exposed to in their diet, daily habits and professional life; 4) participants’ perception of the impact of these factors on their fertility and their desire to obtain medical help to modify such exposure if present.

During the consultation, the risk factors of reprotoxic exposure (within the past 6 months) of each patient was estimated with another questionnaire designed upon two previously published standardized questionnaires used in subfertile patients populations [22, 23]; using this questionnaire, the physician collected qualitative information about profession type, exposure to active or passive smoking, alcohol consumption, physical and chemical reprotoxic agents at home (indoor air, diet, cosmetics, pesticides) or workplace. If this questionnaire detected one or more risk factor of reprotoxic exposure (RFRE), the patient was considered “at risk”.

Finally, we analyzed the correlation between patients’ perceived RFRE and their RFRE estimated by the standardized questionnaire completed by the physician during the consultation. Our aim was to establish whether medical intervention to estimate these risks would be useful in routine practice, or whether patients estimate them well on their own.

### Statistical analysis

The quantitative data were extracted from the questionnaires and analyzed using Excel (Microsoft, Redmond, WA, USA). To compare the answers from each group, the chi-square test of independence and homogeneity and Fisher’s exact test were used. A *p* value < 0.05 was considered statistically significant.

## Results

### Population

The study was suggested to 646 patients and 390 patients (195 couples) participated (60% participation rate). The median age was 33.9 ± 6.2 years (men: 35.4 ± 7 years; women: 32.4 ± 5 years).

### Information source

More than half of the patients were aware of the existence of reprotoxic agents (*n* = 218, 56%). No significant difference was assessed between men and women (54% vs. 58%, *p* = 0.7).

The sources of information used by the patients are shown in Table 1. The major source of information was the media (40%), followed by the internet (22%) and gynecologists (15%).

**Table 1:** Sources of information on reprotoxic agents used by subfertile patients

Sources of information	Men	Women	Total	***p*** value
**Media**	76/195	80/195	156/390	0.68
	39%	41%	40%	
**Internet**	42/195	45/195	87/390	0.71
	21%	23%	22%	
**Occupational physician**	13/195	8/195	21/390	0.26
	7%	4%	5%	
**General practitioner**	20/195	21/195	41/390	0.87
	10%	11%	10%	
**Gynecologist**	19/195	38/195	57/390	0.006
	10%	19%	15%	
**Urologist**	6/195	2/195	8/390	0.15
	3%	1%	2%	
**Andrologist**	2/195	0/195	2/390	0.15
	1%	0%	1%	
**Fertility specialist**	13/195	12/195	25/390	0.84
	7%	6%	6%	

### Evaluation of the patients’ knowledge

The rate of correct answers to the questions assessing knowledge of reprotoxic agents was 61% (6919/11,310). There was no significant difference between men and women (61.3% vs. 61.1%, *p* = 0.8).

Table 2 shows the rate of correct answers given by subfertile patients concerning reprotoxic agents related to diet, daily habits and work.

**Table 2:** General knowledge of subfertile patients concerning dietary, daily life and occupational reprotoxic agents

	Men Number of correct answers / Number of total answers (%)	Women Number of correct answers / Number of total answers (%)	Total Number of correct answers / Number of total answers (%)	***p***-value
**Questions on dietary reprotoxic agents**	952/1560	971/1560	1923/3120	*p* = 0.51
	61%	62%	62%	
**Questions on daily life reprotoxic agents**	1262/1950	1250/1950	2512/3900	*p* = 0.71
	65%	64%	64%	
**Questions on occupational reprotoxic agents**	1240/2145	1244/2145	2484/4290	*p* = 0.93
	58%	58%	58%	
**Total**	3454/5655	3465/5655	6919/11310	*p* = 0.85
	61%	61%	61%	

Detailed questions are presented in part Q5 of the Additional file 1. Detailed answers are presented in Supplementary Data

Concerning reprotoxic agents related to daily habits, extended sitting period was the least recognized male reprotoxic agent (18% of answers were correct, Supplementary data). The best recognized agents were tobacco smoking (88%) and marijuana consumption (81%). Factors that had mixed recognition were overweight (55%), use of detergents and painting products (64%) and fumes released from cars (59%).

Regarding professional reprotoxic agents, exposure to vibration, cement and excessive heat were the least recognized, with correct response rates of 13, 24, and 27%, respectively. The reprotoxic effects of heavy metals such as lead, mercury and cadmium were the most well-known (65%), followed by the effects of solvents (67%), gases (63%), pesticides (68%), ionizing radiation (63%) and motor fuel (63%).

Knowledge of reprotoxic agents related to daily habits and diet tended to be better than knowledge of professional exposure (64 and 62% vs. 58%, *p* = 0.71).

### Opinions of patients concerning the impact of reprotoxic agents on their fertility

The majority of patients (*n* = 203, 52%) considered that decreased exposure to reprotoxic agents could ameliorate their fertility (59% of male vs. 46% of female, *p* = 0.02), while 11% stated that there would be no impact and 36% did not know.

### Desire of patients to obtain medical help to modify exposure to reprotoxic agents

Thirty-eight percent of patients desired medical help or assistance to modify the impact of any exposure (34% of men vs. 41% of women, *p* = 0.14), compared to 35% who did not. In addition, 27% had no opinion on this matter.

### Correlation between risk factor of reprotoxic exposure estimated with the questionnaire and exposure perceived by the patients

In total, 141 patients thought that they had been exposed to reprotoxic agents: 40% of men and 32% of women.

Table 3 shows the individual perception of exposure by the patients and its correlation with the risk factor of reprotoxic exposure (RFRE) estimated with the standardized questionnaire. The questionnaire identified RFRE in 265 patients (68%); risk factor was statistically more frequent in men (77%) than men (59%) (*p* < 0.05).

**Table 3:** Individual perceptions of exposure to reprotoxic agents and the correlation with the risk factor of reprotoxic exposure estimated by the standardized questionnaire

	Nb of patients (%)	Nb of patients (%)	Nb of patients (%)	Total
**Data from the questionnaire before the consulation**	**Perceived exposure**	**Do not know**	**No perceived exposure**	
	141 (36%)	167 (43%)	82 (21%)	390
**Data from the standardized questionnaire used during the consultation**	**Estimated RFRE**	**Estimated RFRE**	**Estimated RFRE**	
	112/141	103/167	50/82	265
	(79%)	(62%)	(61%)	

*Nb* Number, *RFRE* Risk factor of reprotoxic exposure

In total, 141 of the 265 exposed patients (53%) were aware of their RFRE.

## Discussion

The impact of exposure to environmental reprotoxic agents – some of which is modifiable – on the results of assisted reproduction treatment (ART) is increasingly acknowledged. The implementation and efficacy of behavioral changes that can alter such exposure are dependent on the knowledge and perceptions of patients. Our objective was to interrogate our subfertile patients concerning reprotoxic exposure. Half of our population was familiar with the topic. Their knowledge was generally obtained from the media and the Internet. The impacts of alcohol consumption, tobacco smoking and marijuana use were the most well-known. Professional exposure tended to be less commonly known.

We observed a major discrepancy between the risk factor of reprotoxic exposure estimated by the physician and patients’ awareness of their reprotoxic exposure.

### Subfertile patients’ underestimation of their risk factor of exposure to reprotoxic agents

There are many existing studies on the knowledge and perceptions of women concerning their exposure to teratogenic agents during pregnancy, reporting a low level of knowledge [24–28]. However, there is no corresponding data on men and couples in general, especially subfertile ones, besides the pilot study of Christiaens et al. [19] suggesting that most of subfertile men were unaware of their exposure to environmental reprotoxic agents.

Notably, our study indicated that the majority of subfertile patients undergoing ART had risk factors of reprotoxic exposure potentially affecting their fertility, and only half of them were aware of this risk factor of exposure. Their perception did not always correlate with the estimation assessed by the physician, using the standardized questionnaire.

### Nonmedical sources of information about reprotoxic agents

The use of the internet by pregnant women has been previously reported [25, 29, 30]: in this population, the internet was considered the easiest and fastest route to find information about exposure to teratogenic agents during pregnancy [30]. This is relevant primarily for women with a high socioeconomic status [25, 29, 30]. Our previous [23] and current results show that healthcare professionals are not the primary source of information. Consistent with this, our group also previously demonstrated that in our region, healthcare professionals rarely interrogate patients about their exposure, to avoid increasing their stress or because of their inability to provide an appropriate solution [15]. In addition, female patients tend not to discuss information obtained on the internet with their caregiver unless specifically asked [31]. Therefore, healthcare professionals can be largely unaware of incorrect/unsuitable information their patients have obtained from the internet.

### Implications for patients

In our study, more than one patient in three judged essential to decrease their exposure to improve their fertility. These results indicate that subfertile patients are willing to make behavioral changes. This same conclusion was drawn in the International Fertility Decision-Making Study [20], in which women who were interested in fertility modified their daily habits to optimize their pregnancy chances, especially those with elevated BMI and tobacco smokers. Other factors associated with a high desire of subfertile women to reduce their exposure were medical factors (decreased ovarian reserve), education (preconception directives) and financial factors [21]. Regarding subfertile men, we previously suggested that the most common reasons for exposure modification were related to semen parameters impairment and known professional exposure [19].

Notably, in our study, 35% of subfertile patients did not intend to ask for medical help to reduce their environmental exposure, which may be due to the reluctance of patients to declare and discuss their fertility problems on their place of work, particularly with their enterprise’s physician. In previous studies, this attitude was associated with a lack of knowledge of the role and the competencies of the enterprise’s physician and uncertainty about information confidentially with respect to patients’ employer [19, 32].

Another implication of our results for patients is the confirmation of an important need for a deeper exploration of men in subfertile couples. This need was highlighted by the latest American Urologist Association and American Society of Reproductive Medicine guidelines for diagnosis of infertility in men, which recommended that “all infertile men [should] be evaluated by specialists in male reproduction” [33] and suggested that “clinicians may discuss risk factors (i.e., lifestyle, medication usage, environmental exposures) associated with male infertility”. The detection and correction of environmental exposures in subfertile men could improve their fecundity, but also their general health, which has been shown to be poorer than health of fertile men [34].

### Limitations

One limitation of our study is the absence of biomarkers to assess reprotoxic exposures. Studies objectifying exposure through specific biomarkers are necessary for the precise determination of patients’ exposure to chemical agents, but as stated before, the methodology and acceptability of this assessment are very difficult in current practice, because almost no reference values are available and the cost is high. Another limitation is the exclusion of patients not fluent in French, which may have resulted in the exclusion of patients with lifestyles and professions entailing greater exposure to reprotoxic agents. We were also unable to eliminate the recall bias related to the use of a self-report questionnaire.

Lastly, the single-center nature of our study could induce a recruitment bias, however, the population size and the rate of participation made our sample representative of subfertile patients undergoing assisted reproduction.

## Conclusion

This study highlights that the majority of subfertile patients, especially men, requiring assisted reproduction treatment present risks factors of exposure to reprotoxic agents, without awareness of this risk factor of exposure. Their level of knowledge about domestic and professional exposure to reprotoxic agents can be improved.

Altogether, we advocate for implementation of reprotoxic risk factor identification early in the course of the care pathway of subfertile men and women, as they are poorly conscious of their exposure and in order to facilitate the establishment of corrective measures to improve the outcomes of assisted reproduction treatments. Exposure to reprotoxic agents is associated to a risk to public health (fertility, development, offspring health), requiring action at the patient, healthcare professional, authority and societal levels.

## Supplementary Information

**Additional file 1.**

**Additional file 2.**

## Data Availability

All data generated or analysed during this study are included in this published article [and its supplementary information files].

## Abbreviations

ART: Assisted reproductive technique
ELFE: Etude Longitudinale Française depuis l’Enfance (French longitudinal study since childhood)
RFRE: Risk factors of reprotoxic exposures
