# Supplementary material for: Subfertile patients underestimate their risk factors of reprotoxic exposure
Source: Basic Clin Androl. 2022 Jul 5;32:11. doi: 10.1186/s12610-022-00161-z (PMC9254517; doi:10.1186/s12610-022-00161-z)
Supplement: Supplementary file 1 — Additional file 1. [file 12610_2022_161_MOESM1_ESM.docx]

**Appendix**

**Questionnaire evaluating the sources of information and the knowledge of infertile patients regarding reprotoxic agents.** *Expected answers: wrong responses are crossed*

**Patient questionnaire: reprotoxic agents**

**Q1 What are your sex and age ?**

**Q2 Have you ever heard about reprotoxic agents?**

□ YES □ NO

**Q3 If yes, what was your source of information?**

□ Media (TV, magazines, etc.)

□ Internet (forums, etc.)

□ Physician at work

□ General practitioner

□ Gynecologist

□ Urologist

□ Andrologist

□ Embryologist

**Q4 Do you think you have been exposed to any reprotoxic agents?**

A In your diet:

□ YES □ NO □ I do not know

if yes, identify the agent:

B In your daily life:

□ YES □ NO □ I do not know

if yes, identify the agent:

C In your workplace:

□ YES □ NO □ I do not know

if yes, identify the agent:

**Q5 According to you, out of the following substances, which are the ones scientifically identified as toxic to fertility?** *One or multiple answers are possible.*

**A. In one’s diet:**

□ Grilled food

□ Smoked food

~~□ Soda~~

□ Alcohol

□ Food heated in plastic containers

□ Anabolic supplements

~~□ Organic fruits and vegetables~~

~~□ Dairy Products~~

**B.** **In one’s daily life:**

□ Gardening with the use of insecticides and pesticides

□ Overweight

□ Smoking

□ Marijuana

□ Extended sitting periods

~~□ Water vapor~~

~~□ Sea bathing~~

□ Frequent use of painting products

□ Fumes from cars

**C. In one’s workplace:**

□ Heavy metals (lead, mercury, cadmium, etc.)

□ Cement

□ Solvents

□ Gases

□ Vibrations

□ Pesticides

□ X-rays

□ Excessive heat

~~□ Excessive cold~~

~~□ Standing up for long hours~~

□ Motor fuels (petrol derivatives)

**Q6 Do you think that decreasing exposure to reprotoxic agents can improve fertility?**

□ YES □ NO □ I do not know

**Q7 Would you desire medical assistance from the CPMA team to modify you exposure to repro-toxic agents if you were exposed?**

□ YES □ NO □ I do not know

**Q8 Until recently, in your infertility course, did you ever consult with any of the following in order to help reduce your exposure?**

□ Occupational physician □ Dietitian

□ Addictologist/tabaccologist □ Other:
