# Supplementary material for: Subfertile patients underestimate their risk factors of reprotoxic exposure
Source: Basic Clin Androl. 2022 Jul 5;32:11. doi: 10.1186/s12610-022-00161-z (PMC9254517; doi:10.1186/s12610-022-00161-z)
Supplement: Supplementary file 2 — Additional file 2. [file 12610_2022_161_MOESM2_ESM.docx]

**Supplementary data**

**Infertile patients’ general knowledge level regarding dietary/lifestyle/occupational reprotoxic agents**

Nb: number

Comparison between men and women was assessed by Chi-square test

|  | **MEN**  **Nb of correct answers (%)** | **WOMEN**  **Nb of correct answers (%)** | **TOTAL**  **Nb of correct answers (%)** | **Comparison M/W**  **p-value** |
| --- | --- | --- | --- | --- |
| **Grilled food** | 45  23.1% | 53  27.2% | 98  25% |  |
| **Smoked food** | 22  11.3% | 31  15.9% | 53  13.6% |  |
| **Soda** | 141  72.3% | 148  75.9% | 289  74.1% |  |
| **Alcohol** | 163  83.6% | 160  81.5% | 323  82.8% |  |
| **Food heated in plastic containers** | 99  50.8% | 105  53.8% | 204  52.3% |  |
| **Anabolic supplements** | 102  52.3% | 97  49.7% | 199  51% |  |
| **Organic fruits & vegetables** | 193  99% | 193  99% | 386  99% |  |
| **Daily products** | 187  95.9% | 184  94.4% | 371  95.1% |  |
| **First part (dietary reprotoxic agents) total** | **952/1560**  **61%** | **971/1560**  **62.2%** | **1923/3120**  **61.6%** | **p=0.5** |
| **Gardening** | 123  63.1% | 110  56.4% | 233  59.7% |  |
| **Overweight** | 107  54.9% | 106  53.8% | 213  54.6% |  |
| **Smoking** | 171  87.7% | 172  87.7% | 343  87.9% |  |
| **Marijuana** | 156  80% | 159  81% | 315  80.8% |  |
| **Extended sitting periods** | 33  16.9% | 38  19.5% | 71  18.2% |  |
| **Water vapor** | 190  97.4% | 186  95.4% | 376  96.4% |  |
| **Sea bathing** | 194  99.5% | 190  97.4% | 384  98.5% |  |
| **Frequent use of painting agents** | 130  66.7% | 121  62% | 251  64.3% |  |
| **Car fumes** | 112  57.4% | 118  60.5% | 230  59% |  |
| **Second part (lifestyle reprotoxic agents) total** | **1262/1950**  **64.7%** | **1250/1950**  **64.1%** | **2512/3900**  **64.4%** | **p=0.7** |
| **Heavy metals** | 129  66.1% | 126  64.1% | 255  65.4% |  |
| **Cement** | 40  20.5% | 52  26.7% | 92  23.6% |  |
| **Solvents** | 132  67.7% | 130  66.1% | 262  67.2% |  |
| **Gases** | 118  60.5% | 127  65.1% | 245  62.8% |  |
| **Vibrations** | 21  10.8% | 28  14.3% | 49  12.6% |  |
| **Pesticides** | 134  68.7% | 130  66.7% | 264  67.7% |  |
| **X-rays** | 123  63.1% | 121  62% | 244  62.6% |  |
| **Excessive heat** | 53  27.2% | 54  27.7% | 107  27.4% |  |
| **Excessive cold** | 183  94% | 176  90.3% | 359  92% |  |
| **Extended period of sitting** | 181  92.8% | 181  92.82% | 362  92.8% |  |
| **Motor fuels** | 126  65% | 119  61% | 245  62.8% |  |
| **Third part (occupational reprotoxic agents) total** | **1240/2145**  **57.8%** | **1244/2145**  **57.9%** | **2484/4290**  **57.9%** | **p=0.9** |
| **Total** | **3454 /5655**  **61.1%** | **3465/5655**  **61.3%** | **6919/11310**  **61.2%** | **p=0.8** |
